# Supplementary material for: Profiling ubiquitin signalling with UBIMAX reveals DNA damage- and SCFβ-Trcp1-dependent ubiquitylation of the actin-organizing protein Dbn1
Source: Nat Commun. 2023 Dec 14;14:8293. doi: 10.1038/s41467-023-43873-0 (PMC10721886; doi:10.1038/s41467-023-43873-0)
Supplement: Supplementary file 1 — Supplementary Information [file 41467_2023_43873_MOESM1_ESM.pdf]

Supplementary Information for

Profiling ubiquitin signalling with UBIMAX reveals DNA damage- and SCF$^{\beta\text{-TRCP1}}$-dependent ubiquitylation of the actin-organizing protein Dbn1

Camilla S. Colding-Christensen[1†], Ellen S. Kakulidis[1†], Javier Arroyo-Gomez[1], Ivo A. Hendriks[1], Connor Arkinson[2,3], Zita Fábián[1], Agnieszka Gambus[2], Niels Mailand[1], Julien P. Duxin[1*], Michael L. Nielsen[1*]

*Correspondence and requests for materials should be addressed to J.P.D. (email: julien.duxin@cpr.ku.dk) or to M.L.N. (email: michael.lund.nielsen@cpr.ku.dk)

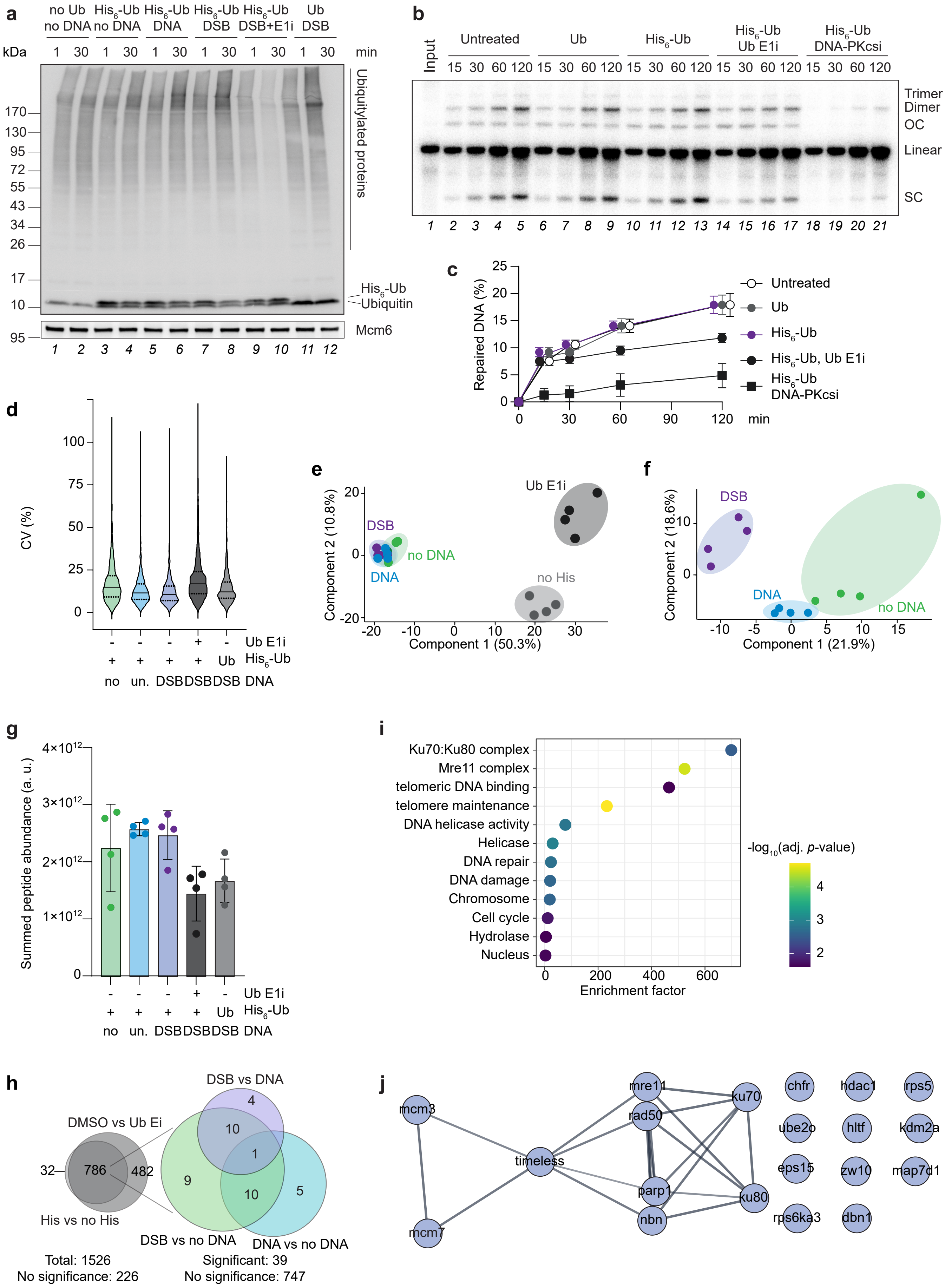
a
no Ub no DNA
His6-Ub no DNA
His6-Ub DNA
His6-Ub DSB
His6-Ub DSB+E1i
Ub DSB
kDa
min
170
130
95
72
55
43
34
26
17
10
Ubiquitylated proteins
His6-Ub
Ubiquitin
Mcm6
1 2 3 4 5 6 7 8 9 10 11 12
b
Input
Untreated
Ub
His6-Ub
His6-Ub Ub E1i
His6-Ub DNA-PKcsi
15 30 60 120
Trimer
Dimer
OC
Linear
SC
1 2 3 4 5 6 7 8 9 10 11 12 13 14 15 16 17 18 19 20 21
c
Repaired DNA (%)
min
Untreated
Ub
His6-Ub
His6-Ub, Ub E1i
His6-Ub DNA-PKcsi
d
CV (%)
Ub E1i
His6-Ub
DNA
no un. DSB DSB DSB
e
Component 2 (10.8%)
Component 1 (50.3%)
Ub E1i
DSB
no DNA
DNA
no His
f
Component 2 (18.6%)
Component 1 (21.9%)
DSB
DNA
no DNA
g
Summed peptide abundance (a. u.)
4×10^12
3×10^12
2×10^12
1×10^12
Ub E1i
His6-Ub
DNA
no un. DSB DSB DSB
h
DSB vs DNA
DMSO vs Ub Ei
32
786
482
His vs no His
4
10
1
9
10
5
DSB vs no DNA
DNA vs no DNA
Total: 1526
No significance: 226
Significant: 39
No significance: 747
i
Ku70:Ku80 complex
Mre11 complex
telomeric DNA binding
telomere maintenance
DNA helicase activity
Helicase
DNA repair
DNA damage
Chromosome
Cell cycle
Hydrolase
Nucleus
Enrichment factor
-log10(adj. p-value)
j
mcm3
mcm7
timeless
mre11
rad50
ku70
parp1
nbn
ku80
chfr
hdac1
rps5
ube2o
hltf
kdm2a
eps15
zw10
map7d1
rps6ka3
dbn1

Supplementary Fig. 1. UBIMAX efficiently and specifically detects ubiquitin-conjugated proteins in response to DSBs. Related to Fig. 1.

a. Western blot analysis of ubiquitin from the experiment in Fig. 1c. **b.** Extracts were untreated or supplemented with ubiquitin E1- (“Ub E1i”) or DNA-PKcs inhibitor (“DNA-PKcsi”) prior to addition of untagged ubiquitin (“Ub”) or His_6-Ubiquitin (“His_6-Ub”) and radioactively labelled, linearized plasmid DNA. DNA was recovered and analysed by agarose gel electrophoresis. Input was radioactively labelled, linearized plasmid DNA alone. SC; supercoiled, OC; open circular. **c.** Quantification of repair products from experiments representatively shown in (b). Error bars represent standard error of the mean. n=4 independent reaction replicates, except for the DNA-PKcsi condition (n=2). **d.** Percent coefficients of variation (CV) calculated across quadruplicate independent reaction replicates of the experiment outlined in Fig. 1d. Full and dotted lines indicate median CVs and 25% and 75% quantiles. no, no DNA; un., undamaged plasmid DNA; DSB, linearized plasmid DNA. **e-f.** Principal component analysis of all samples (**e**) or ubiquitin target-enriched samples (**f**). The principal components displayed represent the greatest degree of variability observed. Eigenvalues indicated on the axes. **g.** Mean summed peptide abundance. Error bars represent standard deviations. a.u., arbitrary units. **h.** Proportionally scaled Venn diagrams showing the overlap of ubiquitylated proteins significantly enriched over controls, presence *versus* absence of His_6-ubiquitin (“His vs no His”) and absence *versus* presence of ubiquitin E1 inhibitor (“DMSO vs Ub E1i”) (left), and the subset of ubiquitylated proteins significantly enriched over controls changing significantly with the presence *versus* absence of undamaged- (“DNA vs no DNA”) or linearized plasmid DNA (“DSB vs no DNA”) or undamaged- *versus* linearized plasmid DNA (“DSB vs DNA”) (right). Gene name annotated isoforms were excluded. Significant enrichments for left and right Venn diagrams were determined by one- and two-tailed Student’s *t*-testing, with permutation-based FDR-control, s0=0.1 and 2500 rounds of randomization, to ensure an FDR≤0.05. **i.** Enrichment analysis of GO terms and Keywords significantly represented (FDR ≤ 0.05) in the “DSB-induced” cluster in Fig. 1h. Significance was determined via two-tailed Fisher’s Exact testing with Benjamini–Hochberg correction for multiple hypotheses testing. **j.** STRING network analysis of ubiquitylated proteins in the “DSB-induced” cluster in Fig. 1h. Source data are provided as a Source Data file.

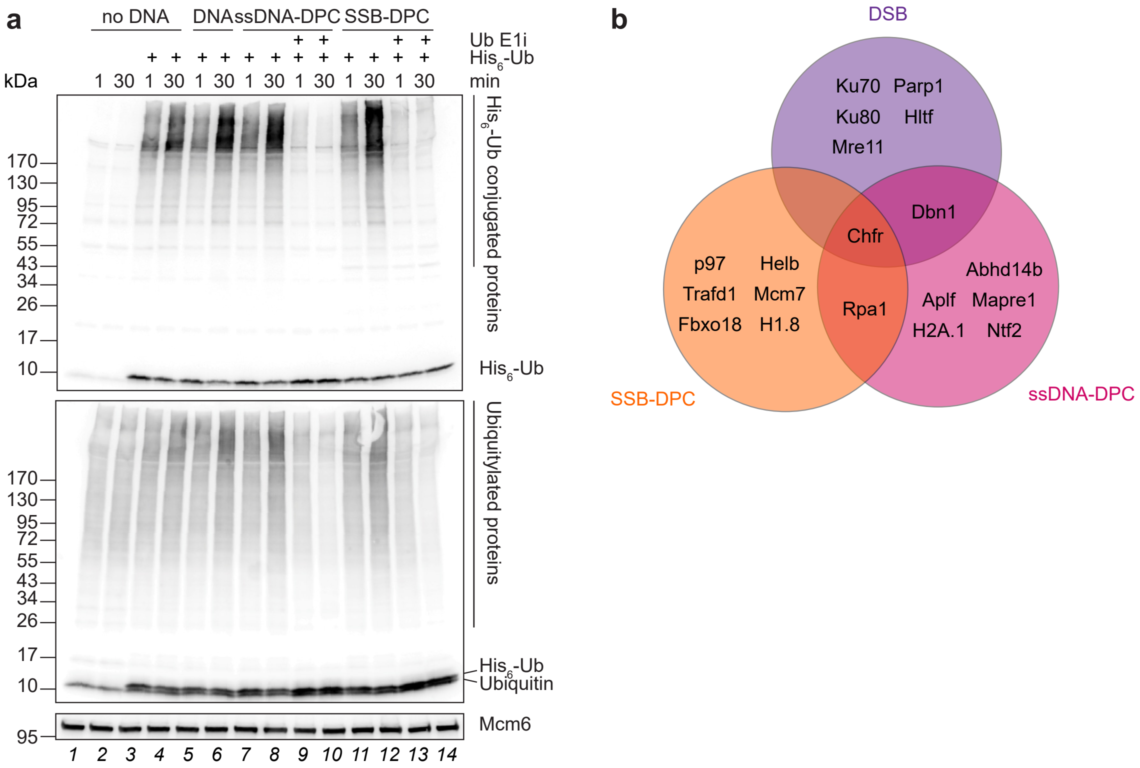
a
no DNA
DNA
ssDNA-DPC
SSB-DPC
Ub E1i
His6-Ub
min
kDa
His6-Ub conjugated proteins
His6-Ub
Ubiquitylated proteins
His6-Ub
Ubiquitin
Mcm6
b
DSB
SSB-DPC
ssDNA-DPC
Ku70
Parp1
Ku80
Hltf
Mre11
Dbn1
Chfr
p97
Helb
Trafd1
Mcm7
Fbxo18
H1.8
Rpa1
Abhd14b
Aplf
Mapre1
H2A.1
Ntf2

Supplementary Fig. 2. UBIMAX identifies DNA damage specific ubiquitylation events. Related to Fig. 2.

a. Extract reactions were performed as described for Fig. 2a, except either in the absence or presence of His_6-ubiquitin (His_6-Ub). Samples were analysed by western blot at 1 or 30 minutes after reaction initiation. Source data are provided as a Source Data file. **b.** Venn diagram showing the overlap of ubiquitylated proteins detected by UBIMAX as significantly enriched in response to linearized plasmid DNA ("DSB"), plasmids carrying the M.HpaII protein crosslinked at a single-stranded DNA gap ("ssDNA-DPC"), or plasmids carrying the Flp protein crosslinked at a single-strand break ("SSB-DPC"), all compared to undamaged plasmid DNA. Only proteins significantly enriched, as determined by two-tailed Student's *t*-testing, with permutation-based FDR-control, with s0=0.1 and 2500 rounds of randomization, to ensure an FDR≤0.01, are shown. n=4 and n=3 independent reaction replicates for the DSB- and DPC-UBIMAX experiments, respectively.

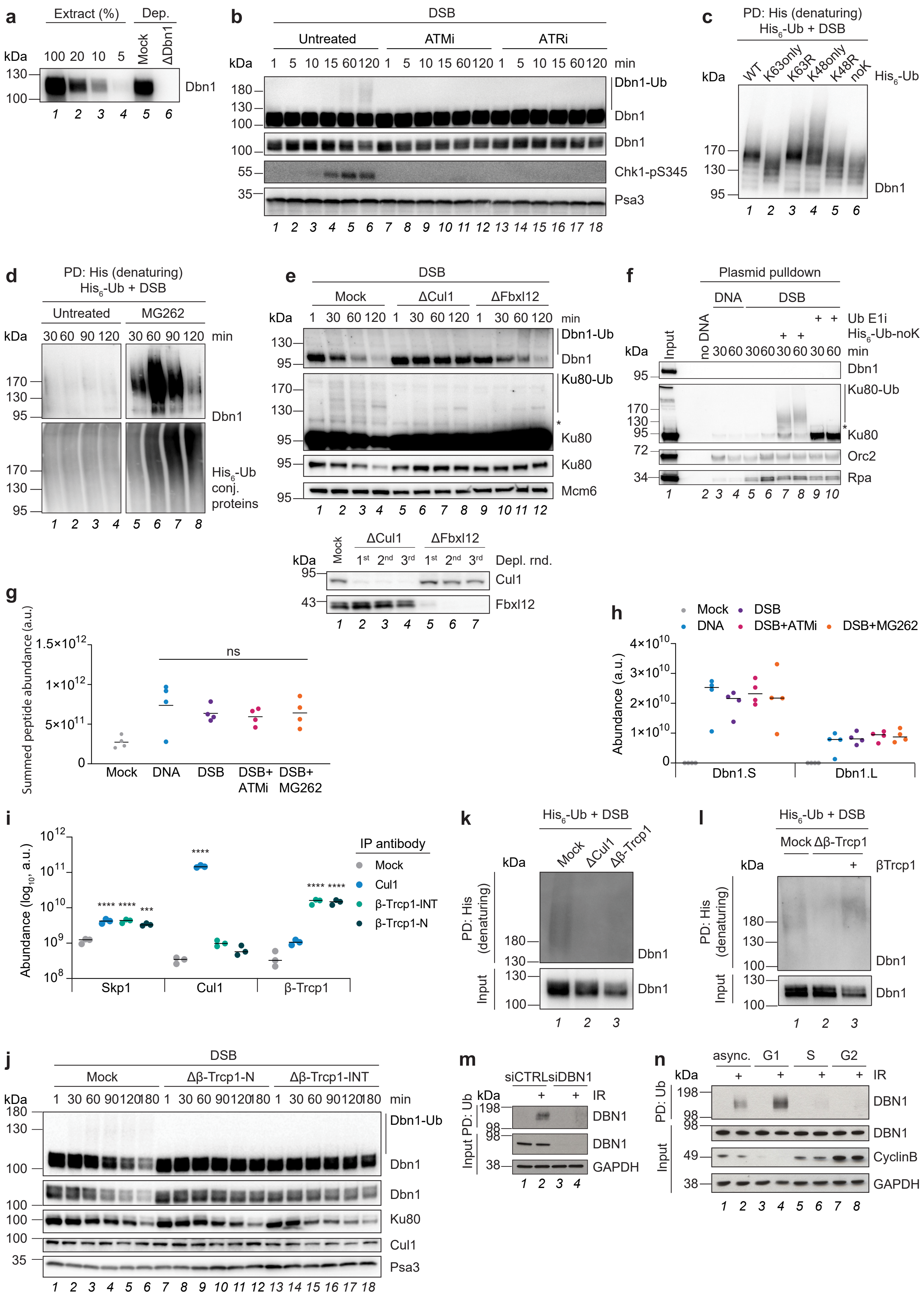
a
Extract (%)
Dep.
kDa
100 20 10 5
Mock
ΔDbn1
130
100
Dbn1
1 2 3 4 5 6
b
DSB
Untreated
ATMi
ATRi
kDa
1 5 10 15 60 120 1 5 10 15 60 120 1 5 10 15 60 120 min
180
130
100
Dbn1-Ub
Dbn1
100
Dbn1
55
Chk1-pS345
35
Psa3
1 2 3 4 5 6 7 8 9 10 11 12 13 14 15 16 17 18
c
PD: His (denaturing)
His6-Ub + DSB
kDa
WT K63only K63R K48only K48R noK
His6-Ub
170
130
95
Dbn1
1 2 3 4 5 6
d
PD: His (denaturing)
His6-Ub + DSB
Untreated
MG262
kDa
30 60 90 120 30 60 90 120 min
170
130
95
Dbn1
170
130
95
His6-Ub conj. proteins
1 2 3 4 5 6 7 8
e
DSB
Mock
ΔCul1
ΔFbxl12
kDa
1 30 60 120 1 30 60 120 1 30 60 120 min
130
95
Dbn1-Ub
Dbn1
170
130
95
Ku80-Ub
*
Ku80
95
Ku80
95
Mcm6
1 2 3 4 5 6 7 8 9 10 11 12
kDa
Mock
ΔCul1
ΔFbxl12
1st 2nd 3rd 1st 2nd 3rd
Depl. rnd.
95
Cul1
43
Fbxl12
1 2 3 4 5 6 7
f
Plasmid pulldown
DNA
DSB
Ub E1i
His6-Ub-noK
kDa
Input
no DNA
30 60 30 60 30 60 30 60 min
95
Dbn1
170
130
95
Ku80-Ub
*
Ku80
72
Orc2
34
Rpa
1 2 3 4 5 6 7 8 9 10
g
Summed peptide abundance (a.u.)
1.5×10^12
1×10^12
5×10^11
0
ns
Mock DNA DSB DSB+ ATMi DSB+ MG262
h
Mock DSB
DNA DSB+ATMi DSB+MG262
Abundance (a.u.)
4×10^10
3×10^10
2×10^10
1×10^10
0
Dbn1.S
Dbn1.L
i
Abundance (log10, a.u.)
10^12
10^11
10^10
10^9
10^8
****
**** **** ***
**** ****
Skp1
Cul1
β-Trcp1
IP antibody
Mock
Cul1
β-Trcp1-INT
β-Trcp1-N
j
DSB
Mock
Δβ-Trcp1-N
Δβ-Trcp1-INT
kDa
1 30 60 90 120 180 1 30 60 90 120 180 1 30 60 90 120 180 min
180
130
100
Dbn1-Ub
Dbn1
100
Dbn1
100
Ku80
100
Cul1
35
Psa3
1 2 3 4 5 6 7 8 9 10 11 12 13 14 15 16 17 18
k
His6-Ub + DSB
kDa
Mock ΔCul1 Δβ-Trcp1
PD: His (denaturing)
180
130
Dbn1
Input
130
100
Dbn1
1 2 3
l
His6-Ub + DSB
Mock Δβ-Trcp1
kDa
+ βTrcp1
PD: His (denaturing)
180
130
100
Dbn1
Input
130
100
Dbn1
1 2 3
m
siCTRL siDBN1
kDa
+ + IR
PD: Ub
198
98
DBN1
Input
98
DBN1
38
GAPDH
1 2 3 4
n
async. G1 S G2
kDa
+ + + + IR
PD: Ub
198
98
DBN1
Input
98
DBN1
49
CyclinB
38
GAPDH
1 2 3 4 5 6 7 8

Supplementary Fig. 3. DDR-dependent and SCF$^{\beta\text{-Trcp1}}$-mediated K48-linked ubiquitylation of Dbn1 results in proteasomal degradation. Related to Fig. 3.

a. Immunodepletion control for Fig. 3a. Dep., depletion. **b.** Western blot analysis of extracts untreated or supplemented with ATM- or ATR inhibitor ("ATMi", "ATRi") prior to addition of linearized plasmid DNA ("DSB"). **c.** Western blot analysis of denaturing His-pulldowns from extracts supplemented with His_6-Ubiquitin ("His_6-Ub") WT or indicated mutants prior to addition of linearized plasmid DNA for 30 minutes. PD, pulldown. **d.** Extracts were untreated or supplemented with proteasome inhibitor (MG262) and His_6-ubiquitin before addition of linearized plasmid DNA and processed as in c. All samples were included on the same membrane and the same exposure is shown. **e.** Western blot analysis of mock-, Cul1- and Fbxl12-immunodepleted extracts upon addition of linearized plasmid DNA. Immunodepletion controls shown below. Depl. rnd, immunodepletion round. *unspecific band. **f.** Western blot analysis of plasmid pulldowns from extracts untreated or supplemented with His_6-ubiquitin-noK ("His_6-Ub-noK") or ubiquitin E1 inhibitor ("Ub E1i") before addition of undamaged- ("DNA") or linearized ("DSB") plasmid DNA. Input was collected before initiation of reaction and unsupplemented extract ("no DNA") was used for the pulldown control. *unspecific band. **g.** Summed peptide abundances of the Dbn1 IP-MS experiment outlined in Fig. 3e. Horizontal lines indicate the median. Error bars represent standard deviations. n=4 independent reaction replicates. Significance was determined by one-way ANOVA with Tukey's multiple comparisons test for all pairwise comparisons. a.u., arbitrary units; ns, not significant. **h.** Abundance of the Dbn1.S and Dbn1.L *Xenopus laevis* isoforms. Horizontal lines indicate the median. a.u., arbitrary units. **i.** Abundance of Skp1, Cul1, and β-Trcp1 from mock-, Cul1-, β-Trcp1-INT-, or β-Trcp1-N-immunoprecipitations (IP) of unstimulated extracts analysed by MS. Horizontal lines indicate the median. Significance was determined by one-way ANOVA with Dunnett's multiple comparisons test for all immunoprecipitations compared to mock, with all indicated comparisons of *p*-value<0.0001 except for Skp1 β-Trcp1-N *versus* mock (*p*-value=0.0007). n=3 independent extract replicates. a.u., arbitrary units. **j.** Western blot analysis of mock-, β-Trcp1-INT-, or β-Trcp1-N-immunodepleted extracts upon addition of linearized plasmid DNA. **k.** Western blot analysis of denaturing His-pulldowns from mock-, Cul1-, or β-Trcp1-immunodepleted extracts supplemented with His_6-ubiquitin and linearized plasmid DNA. Input and pulldown samples were collected prior to and 60 minutes after addition of DNA, respectively. PD, pulldown. **l.** β-Trcp1 protein or buffer was added to mock- or β-Trcp1-immunodepleted extracts prior to His_6-ubiquitin and linearized plasmid DNA. Input and pulldown samples collected and processed as described in (k). **m.** Western blot analysis of whole cell extracts ("input") or ubiquitin pulldowns of siCTRL- or siDBN1-transfected cells 30 min after exposure or not to 10 Gy ionizing radiation (IR). PD, pulldown; Ub, ubiquitin. **n.** Cells were synchronized and released for various times to obtain G1, S or G2 populations before exposure or not to 10 Gy IR and processed as described in (m). Source data are provided as a Source Data file.

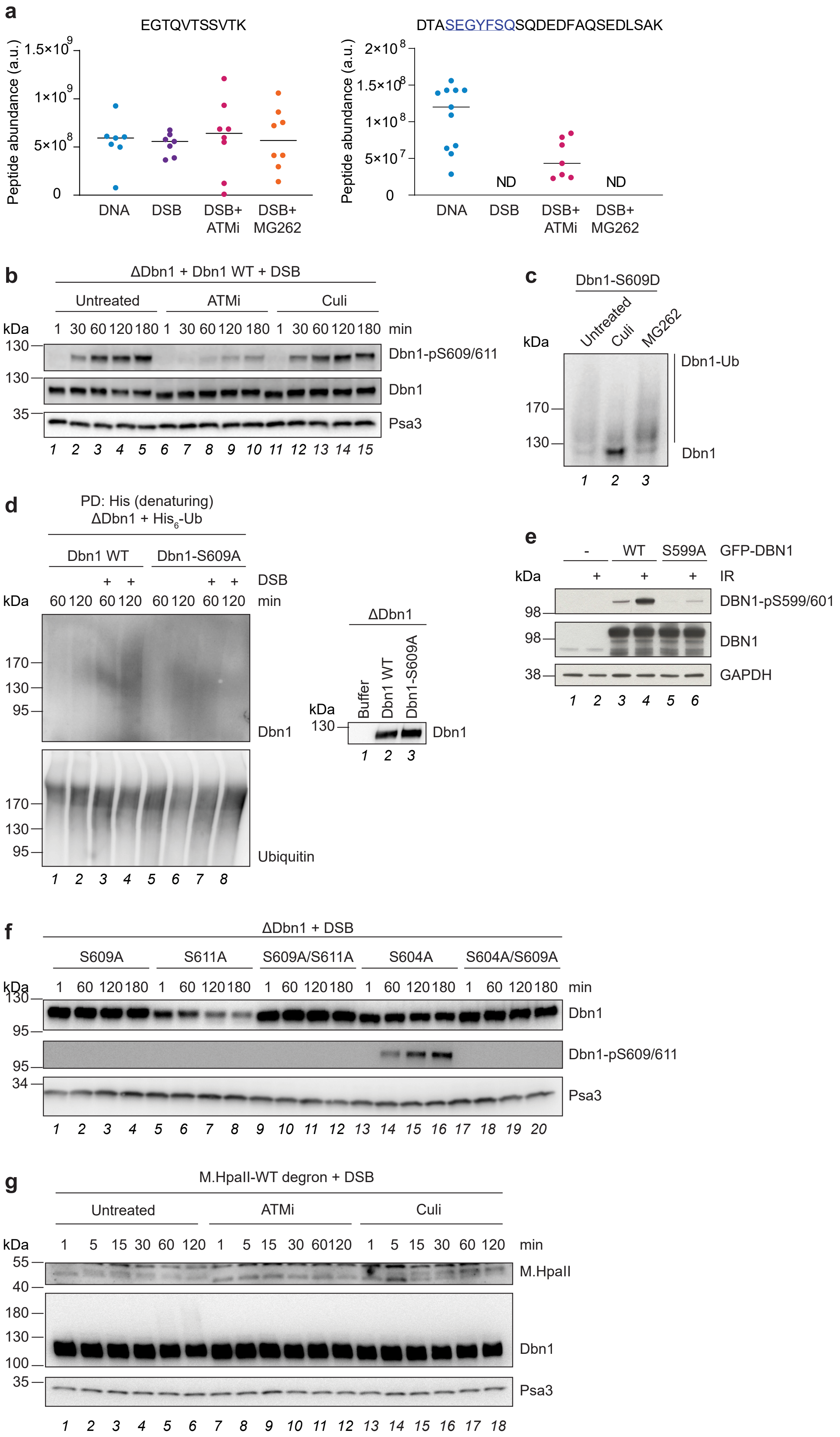
a
EGTQVTSSVTK
DTASEGYFSQSQDEDFAQSEDLSAK
Peptide abundance (a.u.)
DNA
DSB
DSB+ ATMi
DSB+ MG262
ND
b
ΔDbn1 + Dbn1 WT + DSB
Untreated
ATMi
Culi
kDa
min
Dbn1-pS609/611
Dbn1
Psa3
c
Dbn1-S609D
Untreated
Culi
MG262
Dbn1-Ub
Dbn1
d
PD: His (denaturing)
ΔDbn1 + His6-Ub
Dbn1 WT
Dbn1-S609A
DSB
Dbn1
Ubiquitin
ΔDbn1
Buffer
Dbn1 WT
Dbn1-S609A
e
GFP-DBN1
WT
S599A
IR
DBN1-pS599/601
DBN1
GAPDH
f
ΔDbn1 + DSB
S609A
S611A
S609A/S611A
S604A
S604A/S609A
Dbn1
Dbn1-pS609/611
Psa3
g
M.HpaII-WT degron + DSB
Untreated
ATMi
Culi
M.HpaII
Dbn1
Psa3

Supplementary Fig. 4. A variant β-Trcp1 degron is necessary and sufficient for inducing Dbn1 and general protein degradation in response to DSBs. Related to Fig. 4.

a. Abundance of the indicated tryptic Dbn1 peptides across the conditions of the Dbn1 IP-MS experiment outlined in Fig. 3e. Horizontal lines indicate the median. n=4 independent reaction replicates. a.u., arbitrary units; ND, not detected. **b.** Western blot analysis of Dbn1-immunodepleted extracts untreated or supplemented with ATM inhibitor ("ATMi") or neddylation E1 inhibitor ("Culi") prior to addition of Dbn1 protein and linearized plasmid DNA ("DSB"). **c.** Western blot analysis of rabbit reticulocyte lysates (*in vitro* translation system) either untreated or supplemented with neddylation E1 inhibitor or proteasome inhibitor (MG262) before allowing protein translation for 90 minutes initiated by addition of a plasmid encoding Dbn1-S609D. **d.** WT or Dbn1-S609A protein was added to Dbn1-immunodepleted extracts prior to His_6-ubiquitin (His_6-Ub) and undamaged- or linearized plasmid DNA ("DSB"). Samples were collected prior to addition of linearized plasmid DNA for Dbn1-immunodepletion and protein add-back controls (right) and 60 minutes after for denaturing His-pulldown (left). Samples were analysed by western blot. PD, pulldown. **e.** Cells were transiently transfected with control plasmid ("-") or plasmids encoding GFP-DBN1 ("WT") or GFP-DBN1-S599A ("S599A") for 24 hours before being subjected or not to 10 Gy ionizing radiation (IR). Lysates were harvested 30 minutes after irradiation and analysed by western blot. **f.** Western blot analysis of Dbn1-immunodepleted extracts supplemented with the indicated recombinant Dbn1 mutants prior to addition of linearized plasmid DNA. **g.** Western blot analysis of extracts supplemented with M.HpaII protein tagged with the variant β-Trcp1 degron before addition of buffer, ATM-, or neddylation E1 inhibitor and linearized plasmid DNA. Source data are provided as a Source Data file.
