# Supplementary material for: Profiling ubiquitin signalling with UBIMAX reveals DNA damage- and SCFβ-Trcp1-dependent ubiquitylation of the actin-organizing protein Dbn1
Source: Nat Commun. 2023 Dec 14;14:8293. doi: 10.1038/s41467-023-43873-0 (PMC10721886; doi:10.1038/s41467-023-43873-0)
Supplement: Supplementary file 3 — Description of Additional Supplementary Files [file 41467_2023_43873_MOESM3_ESM.pdf]

Description of Additional Supplementary Files:

Supplementary Data 1: UBIMAX in response to DSBs. a. MS analysis of ubiquitylated proteins enriched via the UBIMAX workflow (Fig. 1a) from independent quadruplicate reaction samples and according to the experimental outline in Fig. 1d. Related to Fig. 1d-i, Fig. 2d-i, Supplementary Fig. 1d-j, and Supplementary Fig. 2b. Proteins are enriched from reactions in the presence of "no His", untagged recombinant ubiquitin and linearized plasmid DNA; "Ub E1i", ubiquitin E1 inhibitor, recombinant His6-ubiquitin and linearized plasmid DNA; "no DNA", recombinant His6-ubiquitin; "DNA", recombinant His6-ubiquitin and undamaged plasmid DNA, "DSB", recombinant His6-ubiquitin and linearized plasmid DNA. b. Enrichment analysis of the proteins included in the DSB-induced cluster of the hierarchical clustering analysis shown in Fig. 1h. Related to Supplementary Fig. 1i.

Supplementary Data 2: Total egg extract proteome. Whole proteome MS analysis of biologically independent triplicates of high-speed supernatant interphase egg extracts (HSS). Related to Fig. 1f-g.

Supplementary Data 3: UBIMAX in response to DPCs. MS analysis of ubiquitylated proteins enriched via the UBIMAX workflow (Fig. 1a) from independent reaction triplicates and according to the experimental outline in Fig. 2a. Related to Fig. 2a-i and Supplementary Fig. 2b. Proteins are enriched from reactions in the presence of recombinant His6-ubiquitin and "no DNA", buffer; "DNA", undamaged plasmid DNA, "ssDNA-DPC", plasmids carrying the M.HpaII protein crosslinked at a single-stranded DNA gap; "SSB-DPC", plasmids carrying the Flp protein crosslinked at a single-strand break; "ssDNA-DPC + UbE1i", ubiquitin E1 inhibitor and plasmids carrying the M.HpaII protein crosslinked at a single-stranded DNA gap; "SSB-DPC + UbE1i", ubiquitin E1 inhibitor and plasmids carrying the Flp protein crosslinked at a single-strand break.

Supplementary Data 4: Dbn1 interactome in response to DSBs. MS analysis of proteins (a) and phosphorylation sites (b) enriched via mock- or Dbn1 immunoprecipitation in independent reaction quadruplicates and two technical replicates according to the experimental outline in Fig. 3e. Related to Fig. 3e-h, Supplementary Fig. 3g-h, and Supplementary Fig. 4a. Mock immunoprecipitations were performed in the presence of undamaged plasmid DNA, while proteins immunoprecipitated with Dbn1 antibodies were performed in the presence of "DNA", undamaged plasmid DNA; "DSB", linearized plasmid DNA"; "DSB + ATMi", ATM inhibitor and linearized plasmid DNA; "DSB + MG262", proteasome inhibitor and linearized plasmid DNA.

Supplementary Data 5: MS-based validation of β-Trcp1 antibodies. MS analysis of proteins enriched via mock-, Cul1-, βTrcp1-INT or β-Trcp1-N immunoprecipitations from unstimulated egg extracts. n=3 independent extract replicates. Related to Supplementary Fig. 3i.

Supplementary Data 6: In silico analysis of a DDR-variant β-Trcp1 degron. Proteome-wide sequence analysis of the occurrence of the [DEST]-[DES]-G-x(2)-[ST]-Q variant β-Trcp1 degron across all annotated human proteins. Phosphorylation status of the degron [ST]-Q site, according to the Phospho.ELM and PhosphoSitePlus (PSP) databases, is included. Related to Fig. 4g. HTP, high throughput; LTP, low throughput.
