# Supplementary material for: Profiling ubiquitin signalling with UBIMAX reveals DNA damage- and SCFβ-Trcp1-dependent ubiquitylation of the actin-organizing protein Dbn1
Source: Nat Commun. 2023 Dec 14;14:8293. doi: 10.1038/s41467-023-43873-0 (PMC10721886; doi:10.1038/s41467-023-43873-0)
Supplement: Supplementary file 10 — Reporting Summary [file 41467_2023_43873_MOESM10_ESM.pdf]

nature portfolio

Corresponding author(s): Julien P. Duxin, Michael L. Nielsen

Last updated by author(s): Nov 16, 2023

# Reporting Summary

Nature Portfolio wishes to improve the reproducibility of the work that we publish. This form provides structure for consistency and transparency in reporting. For further information on Nature Portfolio policies, see our Editorial Policies and the Editorial Policy Checklist.

## Statistics

For all statistical analyses, confirm that the following items are present in the figure legend, table legend, main text, or Methods section.

| n/a | Confirmed | |
|---|---|---|
| ☐ | ☒ | The exact sample size ($n$) for each experimental group/condition, given as a discrete number and unit of measurement |
| ☐ | ☒ | A statement on whether measurements were taken from distinct samples or whether the same sample was measured repeatedly |
| ☐ | ☒ | The statistical test(s) used AND whether they are one- or two-sided<br>*Only common tests should be described solely by name; describe more complex techniques in the Methods section.* |
| ☒ | ☐ | A description of all covariates tested |
| ☐ | ☒ | A description of any assumptions or corrections, such as tests of normality and adjustment for multiple comparisons |
| ☐ | ☒ | A full description of the statistical parameters including central tendency (e.g. means) or other basic estimates (e.g. regression coefficient) AND variation (e.g. standard deviation) or associated estimates of uncertainty (e.g. confidence intervals) |
| ☐ | ☒ | For null hypothesis testing, the test statistic (e.g. $F$, $t$, $r$) with confidence intervals, effect sizes, degrees of freedom and $P$ value noted<br>*Give P values as exact values whenever suitable.* |
| ☒ | ☐ | For Bayesian analysis, information on the choice of priors and Markov chain Monte Carlo settings |
| ☒ | ☐ | For hierarchical and complex designs, identification of the appropriate level for tests and full reporting of outcomes |
| ☐ | ☒ | Estimates of effect sizes (e.g. Cohen's $d$, Pearson's $r$), indicating how they were calculated |

*Our web collection on statistics for biologists contains articles on many of the points above.*

## Software and code

Policy information about availability of computer code

| | |
|---|---|
| Data collection | No custom software was used.<br>Western blots were acquired on an Amersham Imager 600 (GE Healthcare) with control software v1.2.<br>Autoradiographs were visualized using a Typhoon FLA 7000 (GE healthcare) with control software v1.3.<br>Mass spectrometry raw data were collected with Thermo Tune v1-1-117-26 and Thermo Xcalibur v4.3.73.11.<br>A list containing the identity, motif sequence and sequence position of the human proteins containing a variant β-Trcp1 degron was generated by submitting the motif [DEST]-[DES]-G-x(2)-[ST]-Q to the ScanProsite tool ((de Castro et al., 2006) to scan against the UniProt Homo Sapiens (taxonomy ID: 9606) database. To this list was mapped the phosphorylation status and kinase relationship of the [ST]-Q site, if known, as retrived from the Phospho.ELM (Diella et al., 2008) and PhosphoSitePlus v6.7.1.1 (Hornbeck et al., 2015) databases. |
| Data analysis | No custom software was used.<br>All mass spectrometry raw data were analyzed using the freely available MaxQuant software (Cox and Mann, 2008), version 1.6.0.1.<br>Quantification and statistical tests of the MaxQuant output files (“proteinGroups.txt”) were performed using Perseus software (Tyanova et al., 2016), version 1.6.0.2, as was Pearson correlation, coefficients of variation, Principal Component, hierarchical clustering, and enrichment analyses.<br>Protein networks were created using the online STRING database, version 11.5 (Szklarczyk et al., 2023).<br>Autoradiographs were quantified using ImageJ, version 1.53t.<br>Graphs and the statistical tests displayed in them were done in Prism (GraphPad), version 9.5.1. |

For manuscripts utilizing custom algorithms or software that are central to the research but not yet described in published literature, software must be made available to editors and reviewers. We strongly encourage code deposition in a community repository (e.g. GitHub). See the Nature Portfolio guidelines for submitting code & software for further information.

## Data

Policy information about availability of data

All manuscripts must include a data availability statement. This statement should provide the following information, where applicable:

- Accession codes, unique identifiers, or web links for publicly available datasets
- A description of any restrictions on data availability
- For clinical datasets or third party data, please ensure that the statement adheres to our policy

All mass spectrometry raw data were analyzed with the Xenopus laevis FASTA database downloaded from Uniprot on the 13th of May 2020 for the total proteome and UBIMAX experiments and on the 3rd of September 2021 for the IP-MS experiments.
The mass spectrometry proteomics data have been deposited to the ProteomeXchange Consortium via the PRIDE partner repository (Perez-Riverol et al., 2022) with the following dataset identifier PXD042086. Source data are provided with this paper.

## Research involving human participants, their data, or biological material

Policy information about studies with human participants or human data. See also policy information about sex, gender (identity/presentation), and sexual orientation and race, ethnicity and racism.

| | |
|---|---|
| Reporting on sex and gender | N/A |
| Reporting on race, ethnicity, or other socially relevant groupings | N/A |
| Population characteristics | N/A |
| Recruitment | N/A |
| Ethics oversight | N/A |

Note that full information on the approval of the study protocol must also be provided in the manuscript.

# Field-specific reporting

Please select the one below that is the best fit for your research. If you are not sure, read the appropriate sections before making your selection.

☒ Life sciences ☐ Behavioural & social sciences ☐ Ecological, evolutionary & environmental sciences

For a reference copy of the document with all sections, see nature.com/documents/nr-reporting-summary-flat.pdf

# Life sciences study design

All studies must disclose on these points even when the disclosure is negative.

| | |
|---|---|
| Sample size | Sample size calculation was not performed.<br>Mass spectrometry experiments were performed in triplicate or quadruplicate (independent egg extracts or egg extract reactions). The size of the individual samples (amount of protein starting material) was determined through pilot experiments.<br>Biochemical analyses (western blots and autoradiographs) were performed at least in duplicate (independent egg extract reactions or independent cell cultures, as applicable). This was considered sufficient based on 1) the reproducibility of results and 2) the consistency of positive and negative controls included in these experiments. |
| Data exclusions | The original analysis of the total proteome included triplicate samples of both HSS and nucleoplasmic extract (NPE). However, as all Xenopus egg extract experiments in this study are otherwise performed in HSS, only this extract was included in the further analysis of the total proteome.<br>The original analysis of the DPC-UBIMAX experiment included four replicates but one replicate was excluded due to significant technical variance.<br>The samples of the Dbn1 IP-MS experiment was aquired as two technical replicates on the basis of C18 StageTip method, with runs resulting from high-pH StageTip clean-up denoted by “H” in the raw files and replicates 01-04 in the analysis, while runs resulting from low-pH StageTip clean-up are denoted by “L” in the raw files and replicates 05-08 in the analysis. Furthermore, this experiment originally included a condition treated with ubiquitin E1 enzyme inhibitor and DSB-mimicking plasmid DNA, but as this condition did not yield significant additional information, it was excluded for further analysis. In figure 3h, only the samples resulting from high-pH StageTip clean up are presented. |
| Replication | Mass spectrometry experiments were performed in triplicate or quadruplicate to ensure reproducibility, as shown by pearson correlation (Fig. 1e), coefficients of variation (Supplementary Fig. 1d), and principal component analysis (Supplementary Fig. 1e-f) for the UBIMAX-DSB experiment. All other MS experiments similarly showed reproducibility.<br>All western blot and autoradiographic analyses were performed at least in duplicate. All attempts at replication were successful.<br>For the experiment shown in Fig. 4c, the reaction containing recombinant Dbn1-S609D was done only once as production of the recombinant protein was possible only in the presence of Cullin inhibitor (Supplementary Fig. 4c). |

| | |
|---|---|
| Randomization | Samples were not divided into experimental groups.<br>All replicates for all individual mass spectrometry experiments were simultaneously prepared, handled, and statistically processed while taking multiple-hypotheses testing into account.<br>Further randomization was not considered relevant for the experimental design of the biochemical analyses of this study. |
| Blinding | All samples relating to each experiment were handled simultaneously. Samples were clearly labeled (and thus not blinded), which is important to both MS, western blot and autoradiographic experimental designs. During handling, all samples were numbered and processed in random order to avoid introduction of bias into the samples.<br>For MS data acquisition, the performance of the MS instrument drifts over time, and there can be power outages and other factors outside of our control. Therefore, it is important to run samples in an order where the least technical variance is introduced between runs (e.g. control_rep1, treatmentA_rep1, treamtentB_rep1, control_rep2, treatmentA_rep2, treatmentB_rep2, etc.). Further, this lets us account for the limited degree of sample carryover as a result from column carryover.<br>All MS data analysis was performed with unbiased software, in an unsupervised manner, and therefore blinding is not applicable in this context. All MS data is publicly available and may be re-processed and investigated by any external party. |

# Reporting for specific materials, systems and methods

We require information from authors about some types of materials, experimental systems and methods used in many studies. Here, indicate whether each material, system or method listed is relevant to your study. If you are not sure if a list item applies to your research, read the appropriate section before selecting a response.

## Materials & experimental systems

| n/a | Involved in the study |
|---|---|
| ☐ | ☒ Antibodies |
| ☐ | ☒ Eukaryotic cell lines |
| ☒ | ☐ Palaeontology and archaeology |
| ☐ | ☒ Animals and other organisms |
| ☒ | ☐ Clinical data |
| ☒ | ☐ Dual use research of concern |
| ☒ | ☐ Plants |

## Methods

| n/a | Involved in the study |
|---|---|
| ☒ | ☐ ChIP-seq |
| ☒ | ☐ Flow cytometry |
| ☒ | ☐ MRI-based neuroimaging |

## Antibodies

| | |
|---|---|
| Antibodies used | Antibodies against Xenopus Mcm6 (Semlow et al., 2016, 1:5000), Orc2 (Fang and Newport, 1993, 1:5000), Rpa (Walter and Newport, 2000, 1:1000) as well as M.HpaII (Larsen et al., 2019, 1:1000) were previously described.<br>The following antibodies were raised against the indicated peptides derived from Xenopus laevis proteins (New England Peptide now Biosynth): Dbn1 (Ac-CWDSDPVMEEEEEEEEGGGFGESA-OH, 1:1000), Ku80 (CMEDEGDVDDLLDMM, 1:1000), Cul1 (H2N-MSSNRSQNPHGLKQIGLDQC-amide, 1:2500), Fbxl12 (Ac-CRGIDELKKSLPNSKVTN-OH, 1:2500), Psa3 (Ac-CKYAKESLEEEDDSDDDNM-OH, 1:5000), β-Trcp1-INT (Ac-GQYLFKNKPPDGKTPPNSC-amide), β-Trcp1-N (H2N-MEGFSSSLQPPTASEREDC-amide), and Dbn1-pS609/611 (Ac-CSEGYF(pS)Q(pS)QDED-amide, 1:2500).<br>The antibodies against His (Fisher Scientific, 631212, lot: 1909019A, 1:1000), Ubiquitin (Santa Cruz, sc-8017, P4D1, lot: B1422, 1:1000), CHK1-pS345 (Cell Signaling, 2341, 133D3, lot: 18, 1:1000), DBN1 (Thermo Fisher Scientific, TA812128 , clone OTI4B1, 1:1000), CUL4A (Cell Signaling, 2699S, lot: 1, 1:1000), GAPDH (Santa Cruz, sc-20357 HRP, lot: G2512, 1:1000) and CYCLIN B (610220, Lot:84924, BD Biosciences, 1:1000) are commercially available.<br>Secondary antibodies used were Peroxidase AffiniPure Goat Anti-Rabbit IgG (H+L) (111-035-003, lot: 156592, Jackson ImmunoResearch, 1:10.000) or Goat Anti-Rabbit IgG Antibody (H+L) (PI-1000, lot: ZJ0211, Vector Laboratories, 1:10.000) and Peroxidase AffiniPure Rabbit Anti-Mouse IgG (H+L) (315-035-003, lot: 127130, Jackson ImmunoResearch, 1:10.000) or Horse Anti-Mouse IgG Antibody (H+L) (PI-2000, lot: ZJ0428, Vector Laboratories, 1:10.000). |
| Validation | References for previously described antibodies raised against Xenopus proteins and M.HpaII are given above.<br>Antibodies raised against Xenopus proteins for this study were validated through their ability to immunoprecipitate and consequently immunodeplete the protein in question from Xenopus egg extract.<br>β-Trcp1-INT and β-Trcp1-N antibodies were validated by immunoprecipitation from Xenopus egg extract followed by mass spectrometry (Supplementary Fig. 3i).<br>All commercially available antibodies were used as per the manufacturer's guidelines and used for the techniques in which they had been validated by the manufacturers.<br>The following antibodies have been validated by the manufacturers for western blotting:<br>His: https://www.fishersci.com/shop/products/6x-his-monoclonal-antibod/NC9720312<br>Ubiquitin: https://www.scbt.com/p/ubiquitin-antibody-p4d1?requestFrom=search<br>CHK1-pS345: https://www.cellsignal.com/products/primary-antibodies/phospho-chk1-ser345-antibody/2341<br>CUL4A: https://www.cellsignal.com/products/primary-antibodies/cul4a-antibody/2699<br>GAPDH: https://www.scbt.com/es/p/gapdh-antibody-v-18<br>DBN1: https://www.thermofisher.com/antibody/product/DBN1-Antibody-clone-OTI4B1-Monoclonal/TA812128<br>CYCLIN B: https://www.bdbiosciences.com/en-us/products/reagents/microscopy-imaging-reagents/immunofluorescence-reagents/purified-mouse-anti-cyclin-b.610220 |

## Eukaryotic cell lines

Policy information about cell lines and Sex and Gender in Research

| | |
|---|---|
| Cell line source(s) | HeLa cells were obtained from ATCC (catalog no. CCL-2). |
| Authentication | The cell lines were not authenticated. |
| Mycoplasma contamination | The cell line used in this study was routinely tested negative for Mycoplasma contamination. |
| Commonly misidentified lines (See ICLAC register) | No commonly misidentified cell lines were used in this study. |

## Animals and other research organisms

Policy information about studies involving animals; ARRIVE guidelines recommended for reporting animal research, and Sex and Gender in Research

| | |
|---|---|
| Laboratory animals | Egg extracts were prepared using eggs (oocytes) collected from mature female Xenopus laevis frogs (> 9 cm) (Nasco Cat #LM0053MX). |
| Wild animals | This study did not involve wild animals. |
| Reporting on sex | Xenopus laevis frogs were solely used for the preparation of egg extract in this study. As only female frogs can produce and lay eggs, the use of male frogs were not considered for this study. Sex was assigned by the vendor (Nasco). |
| Field-collected samples | This study did not involve samples collected in the field. |
| Ethics oversight | All experiments involving animals were approved by the Danish Animal Experiments Inspectorate and are conform to relevant regulatory standards and European guidelines. |

Note that full information on the approval of the study protocol must also be provided in the manuscript.
